# Effects of Imagination on Health

**Published:** 1854-11

**Authors:** 


### HALL'S JOURNAL OF HEALTH.

VOL. I.] NOVEMBER, 1854. [NO. XI.

### EFFECTS OF IMAGINATION ON HEALTH.

WHAT is the nature of that mysterious bond of connection between mind and body we may never know, but the notice of the effects is sometimes interesting, startling, awful, as will be subsequently shown by well attested facts. The general lesson which I wish to inculcate, because of its bearing on the health and happiness of men, is the importance, practically, of keeping the mind constantly employed in something useful and agreeable. One of the great secrets of human happiness is to be profitably busy. Of all men, they are the most miserable, who have nothing to do; and yet, as far as my observation has extended, those who have nothing to do, never have time to do anything. The mechanic who is fully employed, is the very man to perform a job for you punctually. When nothing presses on the attention, the mind is prone to dwell on small things; and strangely too, these small things are, nine times out of ten, among the disagreeables. The absence of a neighborly nod from an acquaintance or fellow-citizen, who never failed to nod before, instantly sets a "nothing-to-do" to work; his whole soul is full of business; so much so, that he can think of nothing else: the mind is tumultuously tossing, and all creation is veiled in a hurting gloom. There is no stopping to inquire whether the offending one is near-sighted; whether he was not going for the doctor, or worse than that, "*shinning it*" among his business friends to meet a note in bank. Mr. *Nothing-To-Do* gets hold of a fact, or story, or occurrence, and by its help he imagines a great wrong has been done him; he pores over it, he cherishes it most pertinaciously, he even wakes up in the night, and thinks about it, until the mind itself is fully roused, and he cannot go to sleep again. The more he thinks, the more sleepless he becomes, and tosses and tumbles about on the bed by the hour; and as the mind becomes hotter, the body begins to sweat, and he gets up in the morning as haggard and weary as an exhausted madman.

A cousin of ours, a Friend, feeling that a kind of an excuse was needed for not taking our Journal, volunteered a reason, that she had taken a "Journal of Health" once, and that a new disease broke out in her family regularly once a month. The plan of the journal appeared to have been, to detail the symptoms of diseases, and with a house full of young Quakeresses, too rich to make it necessary to do any thing, and who did nothing but study how to dart weapons of death and destruction among all the unfortunate singletons about town, notwithstanding their far-famed peace principles, it was an easy thing to spend the spare time in hunting up symptoms.

It is a well known fact among medical men, that a young student of physic will have a dozen different diseases in the first year of his novitiate. Dr. Reese says: "It would seem as if the study of certain diseases sometimes favored their real or imaginary development. (The great Læennec, who spent a large portion of his life in the study of consumption, fell a victim to it himself. So did Wooster, of Cincinnati, and Hastings, of London, who set the world agog on the use of Naptha as a certain cure for Phthisis, and yet he failed to cure himself of it.) Corvisart made disease of the heart his study, and died of it. When the celebrated Professor Frank of Paria was preparing his lectures on disease of the heart, his own became so much disturbed that he was obliged to rest for a while." Men and women have often come to me for the treatment of consumption, when on examination the lungs were found to be as sound and full acting as the lungs of a racehorse, as was afterwards proved by subsequent permanent recovery; a slight thinness in flesh, or pain in the breast, or troublesome cough, from a disordered stomach or liver, or diseased spine, having been magnified to mean that they were falling into a fatal disease. Alas, how often are these imaginings taken vantage of, by wicked men, who have only assumed to be physicians, and subsequent restoration is blazoned abroad, and certified to, in the newspapers as "cures of consumption," when the consumption never existed, but in the imagination of a "*nothing-to-do.*" I often feel, in reference to a patient:—You are too rich to get well; if you had to take in washing at fifty cents a dozen, or had a housefull of children "to do for," and no servant to help, with a sick husband to boot, you would soon be well enough. Let the reader remember, then—

"*A symptom is the very last thing you should think about.*"

It is related of a prime minister, that to prove to his king that actual bodily suffering was less destructive in its influences than imagined danger, he took two lambs, broke the leg of one, placed it in an enclosure with food beside it, and left it; the other, with food beside it, was placed in another enclosure, in which was a tiger, so confined, that he could spring near to the lamb, but could not possibly touch it. Next morning, the wounded lamb had eaten all its food, while that of the other was untouched, and the lamb itself was dead.

Dr. Noble, in an analytic lecture at Manchester, "On the Dynamic Influence of Ideas," told a good anecdote of M. Boutibouse, a French *savant*, in illustration of the power of imagination. M. Boutibouse served in Napoleon's army, and was present at many engagements during the early part of last century. At the battle of Wagram, in 1809, he was engaged in the fray; the ranks around him had been terribly thinned by shot, and at sunset he was nearly isolated. While reloading his musket, he was shot down by a cannon ball. His impression was, that the ball had passed through his legs below his knees, separating them from the thighs; for he suddenly sank down, shortened, as he believed, to the extent of about a foot in measurement. The trunk of the body fell backwards on the ground, and the senses were completely paralyzed by the shock. Thus he lay motionless amongst the wounded and dead during the rest of the night, not daring to move a muscle, lest the loss of blood should be fatally increased. He felt no pain, but this he attributed to the stunning effect of the shock to the brain and nervous system. At early dawn he was roused by one of the medical staff, who came round to help the wounded. "What's the matter with you, my good fellow?" said the surgeon. "Ah, touch me tenderly," replied M. Boutibouse, "I beseech you; a cannon ball has carried off my legs." The surgeon examined the limbs referred to, and then giving him a good shake, said, with a joyous laugh, "Get up with you, you have nothing the matter with you." M. Boutibouse immediately sprang up in utter astonishment, and stood firmly on the legs which he thought he had lost forever. "I felt more thankful," said M. Boutibouse, "than I had ever done in the whole course of my life before. I had not a wound about me. I had, indeed, been shot down by an immense cannon ball; but instead of passing through the legs, as I firmly believed it had, the ball had passed under my feet, and had ploughed a hole in the earth beneath at least a foot in depth, into which my feet suddenly sank, giving me the idea that I had been thus shortened by the loss of my legs." The truth of this story is vouched for by Dr. Noble.

A St. Louis gentleman, who had a slight affection of the head several weeks, became alarmed a few days since, and took the matter so much at heart, that he fully persuaded himself that his head was growing unusually large. It became a settled conviction in his own mind, that it was absolutely swelling. A few nights since, after taking his wife to church, he had occasion to leave and attend a meeting of an association to which he belonged. He was very uneasy while there, occasionally feeling his head, and finally bolted again to the church, to get his wife, and go immediately home. In the hurry of leaving, he picked up another man's hat, vastly too small for him, and in full run, clapped it on his head. What was his horror to find that it wouldn't begin to fit! In vain he tried to press it over his aching brow, but the beaver would'nt yield a particle. This only strengthened his conviction in relation to his growing head, and with the utmost speed he gained the church just as it was breaking up and the people retiring. The congregation were amazed at his absent manner in calling for his wife and then a doctor.

"What is the matter?" said one.

"Oh, matter enough! My head is getting as large as the court-house door—a doctor, quick!"

In a few minutes a physician who was present, came forward, but couldn't satisfy him that his head had no extra bulk. He finally prescribed free bleeding and cupping on the back of his neck. The patient and his wife started home, and called on the way on a cupper and leecher, to get his assistance in the matter.

Just as the man of cups was about to commence operations, the lady observed that her husband had a strange hat, and immediately informed him of the fact. He looked at it carefully for a moment, and his strange fancy of a swelled head seemed to give way under the disclosure, and at once he dispensed with the bloody preparations to reduce it.

Not only the body, but the mind and the heart, become diseased by giving loose to the imagination; in this very way was it, that men were once led into heathenism. Paul states, in the first chapter of Romans, that the old world "became vain in their imaginations, and their foolish heart was darkened," that is, I presume, their judgment was blinded. The reader will also see, I trust, the beautiful appropriateness of scripture language, so often repeated as a caution against "*vain thoughts*," groundless, without reason; these vain imaginations lead to moral and physical death, and ought to be striven against as a religious duty, and that the reader may be aided in the discharge of this duty, I will conclude this article by the narration of an historical incident, well authenticated, and I will venture to say, that when once read, it will never be forgotten, as showing, at least, the power of the imagination.

### "WHO MURDERED DOWNIE?"

About the end of the eighteenth century, whenever any student of the Marischal College, Aberdeen, incurred the displeasure of the humbler citizens, he was assailed with the question, "Who murdered Downie?" Reply and rejoinder generally brought on a collision between "town and gown;" although the young gentlemen were accused of what was chronologically impossible. People have a right to be angry at being stigmatized as murderers, when their accusers have probability on their side; but the "taking off" of Downie occurred when the gownsmen, so maligned, were in swaddling clothes.

But there was a time, when to be branded as an accomplice in the slaughter of Richard Downie, made his blood run to the cheek of many a youth, and sent him home to his books, thoughtful and subdued. Downie was sacrist or janitor at Marischal College. One of his duties consisted in securing the gate by a certain hour; previous to which all the students had to assemble in the common hall, where a Latin prayer was delivered by the principal. Whether, in discharging this function, Downie was more rigid than his predecessor in office, or whether he became stricter in the performance of it at any one time than another, cannot now be ascertained; but there can be no doubt that he closed the gate with austere punctuality, and that those who were not in the common hall within a minute of the prescribed time were shut out, and were afterwards reprimanded and fined by the principal and professors. The students became irritated at this strictness; he, in his turn applied the screw at other points of academic routine, and a fierce war soon began to rage between the collegians and the humble functionary. Downie took care that in all his proceedings he kept within the strict letter of the law; but his opponents were not so careful, and the decisions of the rulers were uniformly agaist them, and in favor of Downie. Reprimands and fines having failed in producing due subordination, rustication, suspension, and even the extreme sentence of expulsion had to be put in force; and, in the end, law and order prevailed. But a secret and deadly grudge continued to be entertained against Downie. Various schemes of revenge were thought of.

Downie was, in common with teachers and taught, enjoying the leisure of the short New Year's vacation—the pleasure no doubt being greatly enhanced by the annoyances to which he had been subjected during the recent bickerings—when, as he was one evening seated with his family in his official residence at the gate, a messenger informed him that a gentleman at a neighboring hotel wished to speak with him. Downie obeyed the summons, and was ushered from one room into another, till at length he found himself in a large apartment hung with black, and lighted by a solitary candle. After waiting for some time in this strange place, about fifty figures, also dressed in black, and with black masks on their faces, presented themselves. They arranged themselves in the form of a court, and Downie, pale with terror, was given to understand he was about to be put on his trial.

A judge took his seat on the bench; a clerk and public prosecutor sat below; a jury was empannelled in front; and witnesses and spectators stood around. Downie at first set down the whole affair as a joke; but the proceedings were conducted with such persistent gravity, that, in spite of himself, he began to believe in the genuine mission of the awful tribunal. The clerk read an indictment, charging him with conspiring against the liberties of the students; witnesses were examined in due form, the public prosecutor addressed the jury, and the judge summed up.

"Gentlemen," said Downie, "the joke has been carried far enough—it is getting late, and my wife and family will be getting anxious about me. If I have been too strict with you in time past, I am sorry for it, and I assure you I will take more care in future."

"Gentlemen of the jury," said the judge, without paying the slightest attention to this appeal, "consider your verdict; and if you wish to retire, do so."

The jury retired. During their absence the most profound silence was observed; and, except renewing the solitary candle that burnt beside the judge, there was not the slightest movement.

The jury returned and recorded a verdict of Guilty.

The judge solemnly assumed a huge black cap, and addressed the prisoner:

"Richard Downie! The jury have unanimously found you guilty of conspiring against the just liberty and immunities of the students of Marischal College. You have wantonly provoked and insulted those inoffensive lieges for some months, and your punishment will assuredly be condign. You must prepare for death. In fifteen minutes the sentence of the court will be carried into effect."

The judge placed his watch on the bench. A block, an axe, and a bag of sawdust, were brought into the centre of the room. A figure more terrible than any that had yet appeared came forward, and prepared to act the part of doomster.

It was now past midnight; there was no sound audible save the ominous ticking of the judge's watch. Downie became more and more alarmed.

"For mercy sake, gentlemen," said the terrified man, "let me home. I promise that you never again shall have cause for complaint."

"Richard Downie," remarked the judge, "you are vainly wasting the few moments that are left you on earth. You are in the hands of those who must have your life. No human power can save you. Attempt to utter one cry, and you are seized, and your doom completed before you can utter another. Every one here has sworn a solemn oath never to reveal the proceedings of this night; they are known to none but ourselves; and when the object for which we have met is accomplished, we shall disperse unknown to any one. Prepare, then, for death; another five minutes will be allowed, but no more."

The unfortunate man in an agony of deadly terror raved and shrieked for mercy; but the avengers paid no heed to his cries. His fevered, trembling lips, then moved as if in silent prayer; for he felt that the brief space between him and eternity was but as a few more tickings of that ominous watch.

"Now!" exclaimed the judge.

Four persons stepped forward and seized Downie, on whose features a cold clammy sweat had burst forth. They bared his neck, and made him kneel before the block.

"Strike!" exclaimed the judge.

The executioner struck the axe on the floor; an assistant on the opposite side lifted at the same moment a wet towel, and struck it across the neck of the recumbent criminal. A loud laugh announced that the joke had at last come to an end.

But Downie responded not to the uproarious merriment—they laughed again—but still he moved not—they lifted him, and Downie was dead!

Fright had killed him as effectually as if the axe of a real headsman had severed his head from his body.

It was a tragedy to all. The medical students tried to open a vein, but all was over; and the conspirators had now to bethink themselves of safety. They now in reality swore an oath among themselves; and the affrighted young men, carrying their disguises with them, left the body of Downie lying in the hotel. One of their number told the landlord that their entertainment was not yet quite over, and that they did not wish the individual that was left in the room to be disturbed for some hours. This was to give them all time to make their escape.

Next morning the body was found. Judicial inquiry was instituted, but no satisfactory result could be arrived at. The corpse of poor Downie exhibited no marks of violence, internal or external. The ill-will between him and the students was known: it was also known that the students had hired apartments in the hotel for a theatrical representation—Downie had been sent for by them; but beyond this nothing was known. No noise had been heard, and no proof of murder could be adduced. Of two hundred students at the college, who could point out the guilty or suspected fifty? Moreover, the students, scattered over the city, and the magistrates themselves had many of their own families amongst the number, and it was not desirable to go into the affair too minutely. Downie's widow and family were provided for—and his slaughter remained a mystery; until, about fifteen years after its occurrence, a gentleman on his death-bed disclosed the whole particulars, and avowed himself to have belonged to the obnoxious class of students who murdered Downie.

